# Como Melhorar os Resultados Clínicos em Mulheres com Fibrilação Atrial

**DOI:** 10.36660/abc.20250095

**Published:** 2025-06-06

**Authors:** Naoya Kataoka, Teruhiko Imamura

**Affiliations:** 1 University of Toyama Toyama Japão University of Toyama, Toyama – Japão

**Keywords:** Anticoagulação, Ablação por Cateter, Acidente Vascular Cerebral

**Ao editor**

A anticoagulação é uma abordagem terapêutica padrão para pacientes com fibrilação atrial (FA), exigindo uma análise cuidadosa do equilíbrio entre a eficácia antitrombótica e o risco de complicações hemorrágicas. Os autores examinaram a taxa de prescrição de anticoagulantes entre pacientes admitidos no pronto-socorro com FA sintomática, revelando uma maior prevalência de não prescrição em mulheres em comparação com homens.^1^ Essa observação levanta diversas preocupações importantes.

O escore mediano CHA_2_DS_2_-VASc foi relatado como 2 em homens e 4 em mulheres,^1^ um valor relativamente modesto. Em contraste, candidatos à oclusão do apêndice atrial esquerdo, tipicamente necessária devido a um risco elevado de sangramento que contraindica a anticoagulação a longo prazo, geralmente apresentam maior escore CHA_2_DS_2_-VASc.^2^ Além disso, estudos recentes sugerem que pacientes com FA assintomática enfrentam maiores riscos de eventos adversos em comparação àqueles com FA sintomática.^3^ A coorte estudada aqui pode não ser totalmente representativa das populações contemporâneas de FA.

O mecanismo subjacente que explica a maior idade de início da FA em mulheres em comparação aos homens permanece obscuro e pode envolver fatores de confusão não abordados.^1^ Por exemplo, o abuso de álcool, um fator de risco conhecido para FA recorrente após ablação por cateter, é supostamente mais prevalente entre os homens,^4^ o que pode ser parcialmente responsável pelas diferenças observadas.

Além disso, os riscos de acidente vascular cerebral e sangramento são significativamente influenciados por intervenções como ablação por cateter e fechamento do apêndice atrial esquerdo. É fundamental esclarecer quantos pacientes no estudo foram submetidos a esses procedimentos e se ocorreram eventos adversos após a intervenção.
