# Supplementary material for: Como Melhorar os Resultados Clínicos em Mulheres com Fibrilação Atrial
Source: Arq Bras Cardiol. 2025 Jun 6;122(6):e20250095. [Article in Portuguese] doi: 10.36660/abc.20250095 (PMC12212388; doi:10.36660/abc.20250095)
Supplement: Translation

# Translation: How to Improve Clinical Outcomes in Women with Atrial Fibrillation

**DOI:** 10.36660/abc.20250095i

**Authors:** Naoya Kataoka, Teruhiko Imamura

**Affiliations:** 1 Japan University of Toyama, Toyama – Japan

**Keywords:** Anticoagulation, Catheter Ablation, Stroke

**To the editor**

Anticoagulation is a standard therapeutic approach for patients with atrial fibrillation (AF), requiring careful consideration of the balance between antithrombotic efficacy and the risk of hemorrhagic complications. The authors examined the rate of anticoagulant prescriptions among patients admitted to the emergency department with symptomatic AF, revealing a higher prevalence of non-prescription in women compared to men.^1^ This observation raises several important concerns.

The median CHA_2_DS_2_-VASc score was reported as 2 in men and 4 in women,^1^a relatively modest value. In contrast, candidates for left atrial appendage closure, typically necessitated by an elevated bleeding risk that contraindicates long-term anticoagulation, generally present with higher CHA_2_DS_2_-VASc scores.^2^ Furthermore, recent studies suggest that patients with asymptomatic AF face higher risks of adverse events compared to those with symptomatic AF.^3^ The cohort studied here may not be fully representative of contemporary AF populations.

The underlying mechanism explaining the higher age of AF onset in women compared to men remains unclear and may involve unaddressed confounding factors.^1^ For example, alcohol abuse, a known risk factor for recurrent AF following catheter ablation, is reportedly more prevalent among men,^4^ which could partly account for the observed differences.

Additionally, the risks of both stroke and bleeding are significantly influenced by interventions such as catheter ablation and left atrial appendage closure. It is imperative to clarify how many patients in the study underwent these procedures and whether adverse events occurred post-intervention.
