# Supplementary material for: Como Melhorar os Resultados Clínicos em Mulheres com Fibrilação Atrial
Source: Arq Bras Cardiol. 2025 Jun 6;122(6):e20250095. [Article in Portuguese] doi: 10.36660/abc.20250095 (PMC12212388; doi:10.36660/abc.20250095)
Supplement: Related texts

# Reply: Carta-resposta

**Authors:** Emiliano Medei, Renata J. Moll-Bernardes, Martha V. Pinheiro, Andréa Silvestre Sousa, Barbara Abufaiad, André Feldman, Guilherme D’andrea Saba Arruda, Thiago Líbano Csernik, Fabio Augusto De Luca, Benhur Henza, Denilson Campos de Albuquerque, Marcia Noya-Rabelo, Antonio Aurelio de Paiva Fagundes, Angelina Camiletti, Rose Frajtag, Ronir Raggio Luiz, Olga Ferreira de Souza

**Affiliations:** 1 Universidade Federal do Rio de Janeiro Rio de Janeiro RJ Brasil Universidade Federal do Rio de Janeiro, Rio de Janeiro RJ – Brasil; 2 Instituto D’Or de Pesquisa e Ensino Rio de Janeiro RJ Brasil Instituto D’Or de Pesquisa e Ensino (IDOR), Rio de Janeiro, RJ – Brasil; 3 Rede D’Or Sao Luiz São Paulo SP Brasil Rede D’Or Sao Luiz, São Paulo, SP – Brasil; 4 Universidade do Estado do Rio de Janeiro Rio de Janeiro RJ Brasil Universidade do Estado do Rio de Janeiro, Rio de Janeiro RJ – Brasil

Os autores apreciam o interesse em nosso artigo e os pontos importantes levantados. Em relação à “representatividade das populações contemporâneas com FA”, concordamos que nossa amostra não é totalmente representativa das populações com FA, conforme afirmamos nas limitações do estudo. Embora tenhamos realizado um estudo multicêntrico com uma amostra muito grande da maior rede hospitalar privada da América Latina, não incluímos hospitais públicos. Além disso, os achados, como os valores de CHA_2_DS_2_VASc (mediana de 2 para homens e 4 para mulheres), foram semelhantes aos do Registro Cardiovascular Brasileiro de Fibrilação Atrial (Estudo RECALL), cuja pontuação média foi de 3,2.^1^

A diferença de idade entre homens e mulheres na população estudada, como já apontado em outros estudos,^2^ pode estar associada a diferentes mecanismos fisiopatológicos. Como mencionado, o consumo de álcool, um conhecido fator de risco para fibrilação atrial, pode ser um fator importante associado ao aumento da incidência de taquiarritmias em homens jovens.^3^

De fato, a ablação e o fechamento do apêndice atrial esquerdo são intervenções que podem reduzir o risco de acidente vascular cerebral e sangramento a longo prazo. No entanto, nosso estudo não se concentrou na prevalência de complicações clínicas associadas à fibrilação atrial, mas sim na adequação da indicação da terapia anticoagulante, revelando resultados paradoxais em relação ao gênero, com mulheres apresentando maior risco, porém menor indicação de profilaxia. Além disso, como não há consenso sobre a descontinuação da terapia antitrombótica após a ablação, esses aspectos não foram incluídos na análise, sem afetar as conclusões do estudo.

## References

[1] Lopes RD, Barros e Silva PGM, Hoffmann CR, Cavalvante MA, Miranda CM, Esper RB (2023). The First Brazilian Cardiovascular Registry of Atrial Fibrillation: Primary Results of the RECALL Study. Am Heart J.

[2] Lee KK, Doudesis D, Bing R, Astengo F, Perez JR, Anand A (2023). Sex Differences in Oral Anticoagulation Therapy in Patients Hospitalized with Atrial Fibrillation: A Nationwide Cohort Study. J Am Heart Assoc.

[3] van Gelder IC, Rienstra M, Bunting KV, Casado-Arroyo R, Caso V, Crijns HJGM (2024). 2024 ESC Guidelines for the Management of Atrial Fibrillation Developed in Collaboration with the European Association for Cardio-Thoracic Surgery (EACTS). Eur Heart J.

# Reply

**Authors:** Emiliano Medei, Renata J. Moll-Bernardes, Martha V. Pinheiro, Andréa Silvestre Sousa, Barbara Abufaiad, André Feldman, Guilherme D’andrea Saba Arruda, Thiago Líbano Csernik, Fabio Augusto De Luca, Benhur Henza, Denilson Campos de Albuquerque, Marcia Noya-Rabelo, Antonio Aurelio de Paiva Fagundes, Angelina Camiletti, Rose Frajtag, Ronir Raggio Luiz, Olga Ferreira de Souza

**Affiliations:** 1 Brazil Universidade Federal do Rio de Janeiro, Rio de Janeiro RJ – Brazil; 2 Brazil Instituto D’Or de Pesquisa e Ensino (IDOR), Rio de Janeiro, RJ – Brazil; 3 Brazil Rede D’Or Sao Luiz, São Paulo, SP – Brazil; 4 Brazil Universidade do Estado do Rio de Janeiro, Rio de Janeiro RJ – Brazil

The authors appreciate the interest in our paper and the important points raised. Regarding the “*representativeness of contemporary AF populations*”, we agree that our sample is not fully representative of AF populations, as we stated in the limitations of the study. Even though we performed a multicenter study with a very large sample from the largest private hospital network in Latin America, we did not include public hospitals. Moreover, the findings, such as the CHA_2_DS_2_VASc values (median of 2 for men and 4 for women), were similar to those of the Brazilian Cardiovascular Registry of Atrial Fibrillation (RECALL Study), whose average score was 3.2.^1^

The difference in age between men and women in the study population, as already pointed out in other studies,^2^ may be associated with different pathophysiological mechanisms. As mentioned, alcohol use, a known risk factor for atrial fibrillation, may be an important factor associated with the increased incidence of tachyarrhythmia in young men.^3^

Indeed, ablation and left atrial appendage closure are interventions that can reduce the long-term risk of stroke and bleeding. However, our study did not focus on the prevalence of clinical complications associated with atrial fibrillation but rather on the appropriateness of anticoagulant therapy indication, revealing paradoxical results related to gender, with women exhibiting a higher risk yet lower prophylaxis indication. Furthermore, since there is no consensus on discontinuing antithrombotic therapy after ablation, these aspects were not included in the analysis without affecting the study’s conclusions.
